# Peter Reddaway, PhD

**DOI:** 10.1192/bjb.2024.105

**Published:** 2025-08

**Authors:** Robert van Voren

Formerly Professor Emeritus of Political Science and International Relations, George Washington University, USA

**Figure:**
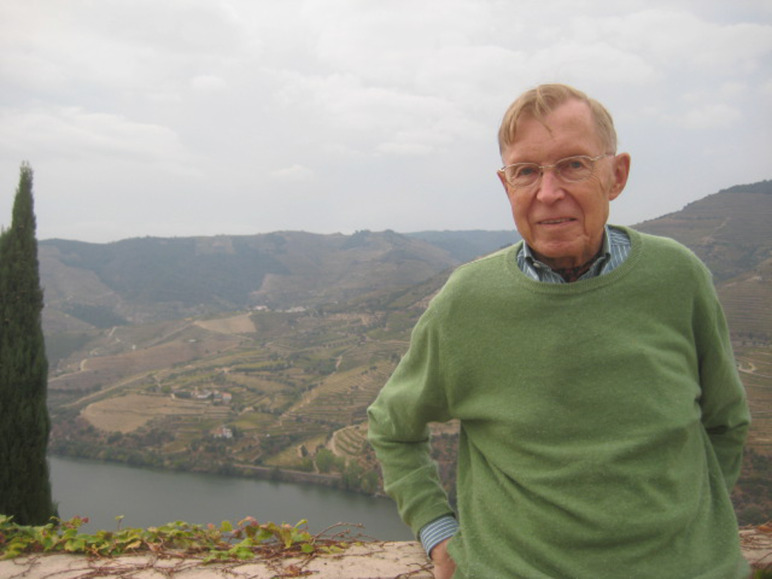

At the end of May 2024, the last two dozen boxes with archives and books belonging to Professor Peter Reddaway arrived in Lithuania. He died 2 months later, 28 July 2024, at the age of 84. Over the past years his complete library of some 9000 books and more than 30 metres of archives found their way to the Lithuania-based Andrei Sakharov Research Center for Democratic Development. They represented a unique collection of documents of one of the most prominent Sovietologists in the post-war period and a central figure in the Western support for the human rights movement in the USSR. However, Peter Reddaway will probably be most remembered for his key role in starting the campaign against political abuse of psychiatry in the USSR.

Peter Reddaway was born in Cambridge on 18 September 1939. His father, Brian Reddaway, was Professor of Political Economy at the University of Cambridge. His mother, Barbara Bennett, was a physiotherapist. After finishing school, he studied Russian at King's College, Cambridge and after graduation won a scholarship to study at Moscow State University. However, he was expelled from the USSR after meeting the wife of a Soviet defector to the UK. He subsequently enrolled in the London School of Economics for a PhD in Soviet studies and taught there until 1986. In 1972 Reddaway married Kathleen Teitgens. They had two children and divorced in 1987. Two years later he married Elizabeth (Betsy) Burton.

By the end of the 1960s Peter Reddaway had become one of the key organisers of support in the West. In 1968 he established the Herzen Foundation with the Leyden Professor Karel van het Reve, organising the publication of many Soviet banned dissident publications in the West and smuggling materials to the Soviet Union. The political abuse of psychiatry, which had become one of the main tools to fight the ‘internal enemy’ when Yuri Andropov became Director of the KGB in 1967, caught Peter's eye. He tried to bring this perversion of medical practice to the attention of the World Psychiatric Association (WPA). In 1971 the WPA refused to even discuss the matter, but in 1977 he succeeded, with his fellow campaigners, to have the political abuse condemned by the General Assembly of the WPA. In the same year, with Sidney Bloch, he published *Russia's Political Hospitals*, the first detailed account of the system of abuse published in the West. A second book, *Soviet Psychiatric Abuse: The Shadow over World Psychiatry*, followed in 1983.

I met Peter for the first time in 1978, introduced to him by the Soviet dissident Vladimir Bukovsky, who had been released less than 2 years earlier after serving in total 12 years in camps, prisons and psychiatric hospitals. The introduction to Peter Reddaway changed the course of my life. I was barely 19, having just left secondary school, but somehow both Bukovsky and Reddaway were convinced I could be useful to the campaign to support the dissident movement in the USSR. Quite soon Peter became a mentor, introducing me to the world of couriers who travelled to the USSR to deliver humanitarian aid to the dissident movement and smuggle out documents and unofficial literature. My first visit was in early 1980, and over the next 9 years I made some 40 visits. Initially I was one of Peter's ‘team members’, but I gradually developed my own ‘team’, recruiting and instructing couriers on how to smuggle information and documents out of the country undetected. With Peter and several others, we exchanged the information obtained, and thus fuelled the campaign for the release of political prisoners. Peter's position as both an academic and a very active human rights defender was unique.

In 1980 we were both among the founders of the International Association on the Political Abuse of Psychiatry (IAPUP), which is now the Federation Global Initiative on Psychiatry (FGIP). His role was central. He collected all the evidence from a wide range of sources, including information brought back by our couriers, and then developed endless strategies to convince national psychiatric associations to pressure the WPA to have the Soviet member society expelled. In 1983 the Soviets withdrew to avoid expulsion. We then aimed to prevent a return to the WPA of the Soviets as long as the political abuse continued. During the years 1977–1989 the Royal College of Psychiatrists played a central role in this campaign. Peter's detailed reports and constant flow of information to the College was instrumental in maintaining the pressure. Kenneth Rawnsley and Jim Birley, both past Presidents of the College, became close friends and supporters, and it is no coincidence that Jim Birley later became Chairman of FGIP.

After moving to the USA in 1986, Peter directed the Wilson Center's Kennan Institute for Advanced Studies in Washington DC (1986–1989) and then taught at George Washington University (1989–2004), retiring as Professor Emeritus of Political Science and International Relations. However, he remained active till the last moment. Like many of us Sovietologists, he was deeply saddened by the fact that Russia returned to Stalinism and felt that all our investments in a democratic Russia had been in vain. Over the past years we corresponded frequently about the resumption of political abuse of psychiatry in Russia and how to bring this again to the attention of the world psychiatric community.

Peter Reddaway was a giant, a central figure in the campaign to bring the political abuse of psychiatry in the USSR to an end and one of the most humble and humane persons I have known. He will be sorely missed by his wife Betsy and children Christopher and Rebecca.

